# Nanoscale LiZnN - Luminescent Half-Heusler Quantum Dots

**DOI:** 10.1021/acsaom.3c00065

**Published:** 2023-06-06

**Authors:** S. Carter-Searjeant, S. M. Fairclough, S. J. Haigh, Y. Zou, R. J. Curry, P. N. Taylor, C. Huang, R. Fleck, P. Machado, A. I. Kirkland, M. A. Green

**Affiliations:** † Department of Physics, King’s College London, Strand, London WC2R 2LS, U.K.; ‡ Department of Materials, University of Manchester, Oxford Road, Manchester M19 9PL, U.K.; § Department of Electrical and Electronic Engineering, Photon Science Institute, University of Manchester, Oxford Road, Manchester M13 9PL, U.K.; ∥ Sharp Life Science (EU) Ltd., The Hayakawa Building, Edmund Halley Road, Oxford Science Park, Oxford OX4 4GB, U.K.; ⊥ Electron Physical Sciences Imaging Centre, Diamond Light Source, Harwell Science Innovation Campus, Fermi Ave, Didcot OX110DE, U.K.; # Centre for Ultrastructural Imaging, King’s College London, New Hunts House, Guys Campus, London SE1 1UL, U.K.; ¶ Department of Materials, University of Oxford, Parks Road, Oxford OX1 3PH, U.K.

**Keywords:** quantum dots, half-Heusler, nitrides, Nowotny-Juza, heavy metal free

## Abstract

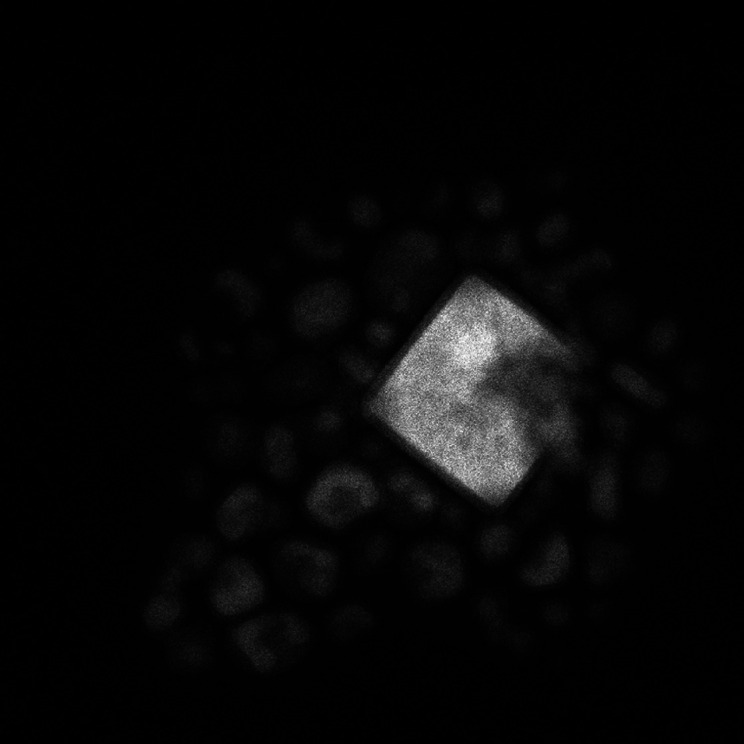

Colloidal semiconductor quantum dots are a well-established technology, with numerous materials available either commercially or through the vast body of literature. The prevalent materials are cadmium-based and are unlikely to find general acceptance in most applications. While the III–V family of materials is a likely substitute, issues remain about its long-term suitability, and other earth-abundant materials are being explored. In this report, we highlight a nanoscale half-Heusler semiconductor, LiZnN, composed of readily available elements as a potential alternative system to luminescent II–VI and III–V nanoparticle quantum dots.

## Introduction

In the search for environmentally acceptable nanoscale luminescent particles, numerous candidates have been explored.^1,2^ Recently, zinc nitride (Zn_3_N_2_), a semiconducting material with an indeterminate band gap and an exciton diameter predicted to be 7.5 nm,^3^ has been suggested as a possible next-generation nanomaterial due to the wide spectral emission tuning range observed (from *ca*. 500 nm to *ca*. 1100 nm) when prepared as colloidal quantum dots, the high quantum yields of up to 50%, and the stable constituent elements.^4^ However, the materials were prepared by the thermolysis of ammonia gas and diethylzinc at elevated temperatures under an inert atmosphere, which makes the synthetic process challenging. It is also worth noting that the resulting quantum dots were extremely air sensitive, although this is less of an issue, as most nanoscale semiconductors prepared in solution require a further inorganic shell layer to make the materials suitable for practical applications.

**Figure 1:**
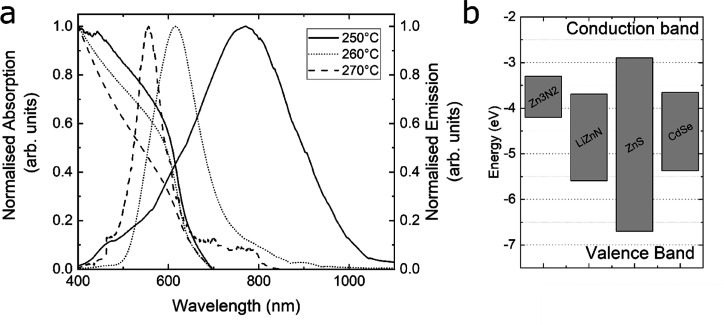
Emission and absorption spectra of Zn_3_N_2_ (50 min reaction time, 250 °C) and the gradual conversion to LiZnN (70 min reaction time, 270 °C). Inset - Literature band alignments of Zn_3_N_2_, LiZnN, ZnS, and CdSe.^12−14^

If one was considering a direct alternative for the prototypical quantum dot material—CdSe—several further factors require consideration (beyond the unclear band gap data and the issues of air sensitivity of Zn_3_N_2_). To deposit a protecting inorganic layer, an appropriately small lattice mismatch is required. For example, a lattice mismatch of *ca*. 11.6% allows the formation of simple CdSe/ZnS core/shell quantum dots. Using the accepted convention, a simple comparison of lattice constants suggests a lattice mismatch of *ca*. 45% for Zn_3_N_2_/ZnS, which is too large for a smooth shell deposition process leading to a spherical particle. It should however be noted that Zn_3_N_2_ has an antibixbyite crystal structure with a distorted octahedral coordination and thus displays symmetries requiring a larger unit cell, and therefore, a simple lattice constant comparison might not be an appropriate measure for determining a likely lattice mismatch. An alternative comparison might be the bond length for Zn–N (2.133 Å)^5^ vs Zn–S (2.0464 Å),^6^ giving a bond length mismatch of between *ca*. 4.23%, which suggests ZnS shelling could be possible. Likewise, the second prerequisite for a shell material that maintains or enhances the optical properties is a suitable band alignment, as shown in Figure 1, inset. While Zn_3_N_2_/ZnS has a band alignment consistent with a type I heterostructure, it is clearly staggered when compared to CdSe/ZnS.

An alternative approach to synthesizing an inorganic coating on the Zn_3_N_2_ surface is to convert the Zn_3_N_2_ itself. Here, we demonstrate the use of Zn_3_N_2_ quantum dots as a scaffold, into which lithium ions can be inserted to produce a related material, the Nowotny-Juza compound LiZnN, as previously described for bulk phase thin films.^7,8^ This was achieved in the colloidal state by choosing relatively air-stable precursors for Zn_3_N_2_ synthesis, which also incorporated lithium (namely LiNH_2_), followed by increasing the synthesis temperature once the intermediary nanoparticulate Zn_3_N_2_ had formed. The resulting phase, LiZnN, was a tetrahedrally filled zinc-blende half-Heusler semiconductor, with a bulk band gap of 1.91 eV,^9^ an excitonic diameter of 9.5 nm,^10^ and a lattice constant of 4.879 Å^3b^ giving a lattice mismatch, *cf*., ZnS of 11% and a band position as shown in Figure 1, inset.^12−14^ Such figures compare favorably with similar values from CdSe (Eg = 1.67 eV, exciton diameter of 11.2 nm, and lattice mismatch with ZnS of 11.6%). It should be noted that several tetrahedrally filled semiconductors have been prepared at the nanoscale through organometallic chemistry, although these have primarily been explored as thermoelectric materials, and few optical properties have been reported.^15−17^

In a typical synthesis, zinc iodide, lithium amide, and hexadecanethiol were mixed in octadecene under an inert atmosphere and gradually heated while stirring. The solution darkened in color to black over a 50 min period with heating up to 250 °C, consistent with the presence of Zn_3_N_2_ quantum dots.^4^ During the reaction, a waste precipitate was observed in the bottom of the reaction flask, and this was eventually discarded. At synthesis temperatures above 250 °C, the black solution changed gradually to dark red due to the insertion of the lithium ion into the lattice of Zn_3_N_2_, forming LiZnN, a material with a different band gap than the scaffold material. The reaction flask could then be removed from the heat source and allowed to cool to room temperature, whereupon the particles present in the supernatant were isolated by solvent/nonsolvent interactions (all experimental details provided in the Supporting Information).

The optical properties of the products are shown in Figure 1. Initial formation of Zn_3_N_2_ quantum dots (reaction temperature of 250 °C, 50 min) was evident by the onset of absorption at *ca*. 650 nm and emission profile in the near-infrared region (*ca*. 750 nm), consistent with the previous report.^4^

A further increase in reaction temperature (to 260 °C) for a further 10 min (total run time 60 min) resulted in a significant blue-shift in the emission profile (to *ca*. 625 nm), the shift continuing to a final emission profile at *ca*. 575 nm (reaction temperature of 270 °C and a total run time of 70 min). Notably, during this period, the absorption band edge position remained essentially unchanged, while the emission profile blue-shifted. This initial blue-shift in emission profile was associated with lithium insertion into the lattice, resulting in LiZnN particles with new structural and electronic characteristics. Inspection of the low-energy tails of the materials synthesized at 260 and 270 °C clearly show extended emission, beyond the onset of absorption, that is characteristic of trap states that are commonly associated with nanocrystal surfaces. It was also observed that the full width half-maximum (fwhm) of the LiZnN emission profile (45 nm) was significantly narrower than for the parent Zn_3_N_2_ material (60 nm) and recently reported InN:Zn (160 nm).^18^ This blue-shift in optical profile from the near-IR to red spectral region is consistent with Li insertion in thin films of Zn_3_N_2_ as described by Moriga et al.^19^ Emission quantum yields were found to be as high as 15% (270 °C, 70 min).

**Figure 2:**
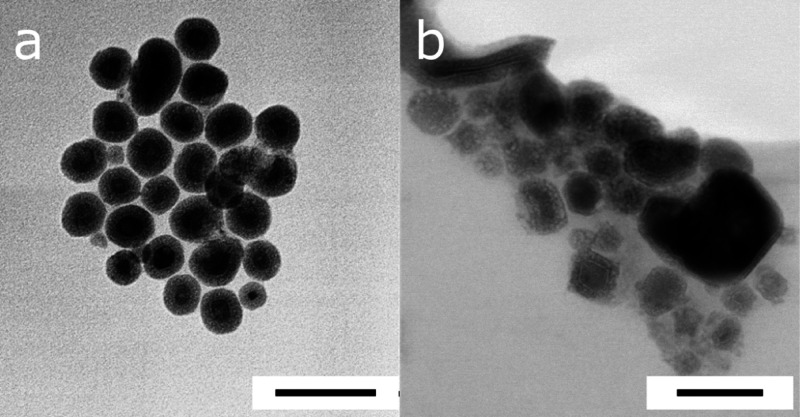
High-angle annular dark-field (HAADF) images of nanoparticles synthesized for 50 min at (a )250 °C and (b) 270 °C, 70 min total growth. Scale bars = 50 nm.

Electron microscopy of samples prepared over a range of temperatures identified faceted nanomaterials with a distinct core/shell morphology. Samples isolated after 50 min/250 °C (Figure 2a, Zn_3_N_2_) were approximately spherical with an average particle size of 22.3 nm with a standard deviation of ±12.3 nm and a shell of lighter contrast material clearly visible (average thickness of up to 10 ± 3 nm). A longer growth time and higher synthesis temperature of up 270 °C did not result in any significant further particle growth, although a small number of larger cuboid particles, up to 200 nm per vertex, which also displayed a shell morphology, were also observed at this point (Figure 2b). It should be noted that the particles described here are overall significantly larger (*ca*. 22 nm) than the Zn_3_N_2_ particles we described in an earlier report (*ca*. 8 nm) yet emit slightly further toward the blue end of the spectrum, consistent with smaller particles. In the current study, we observed a significant oxide shell as shown in Figure 2a, of up to 10 nm in thickness, which means the core Zn_3_N_2_ particles here were roughly a similar size, if not slightly smaller to those reported earlier, and hence emit in a similar, yet slightly blue-shifted spectral position.^20^

**Figure 3:**
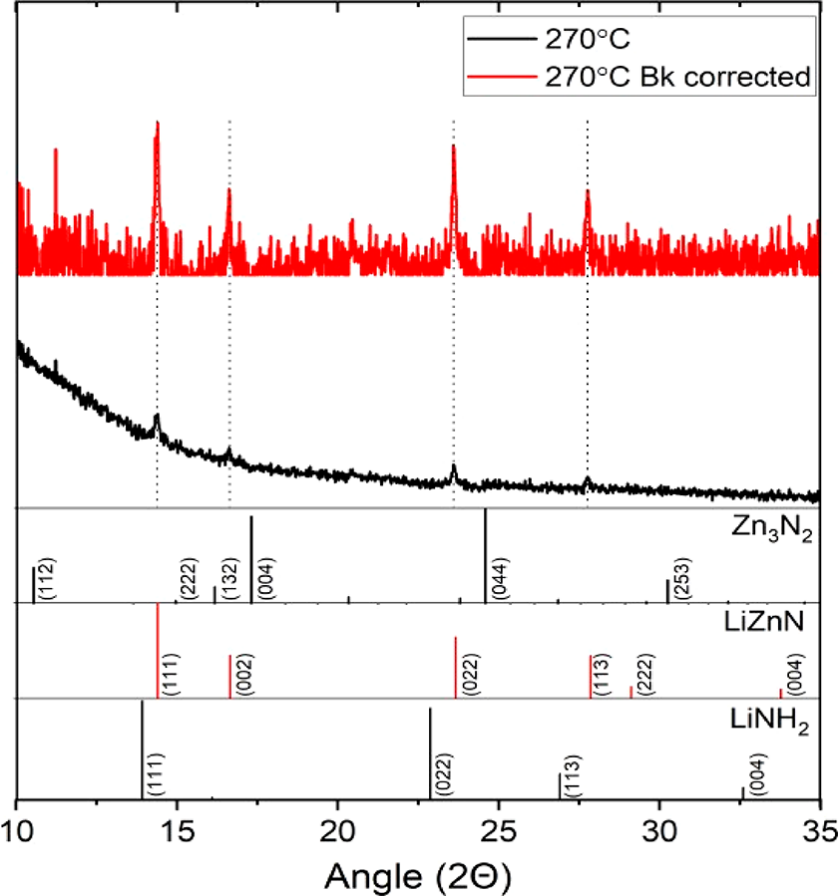
X-ray powder diffraction patterns of nanoparticles prepared at 270 °C/70 min showing the diffraction pattern of LiZnN (Mo radiation). PDF card numbers for the references are provided in the Supporting Information.

X-ray powder diffraction (XRD) analysis of the isolated particles (synthesized at 270 °C) gave a weak diffraction pattern consistent with antifluorite LiZnN, with clear 111, 002, 022, and 113 reflections and with no obvious contribution from crystalline Zn_3_N_2_ or LiNH_2_ (Figure 3).^8^ The weak reflections were also narrow in comparison to the broad reflections normally obtained with the simple cubic III–V family of materials. A brief simulation of 22 nm LiZnN particles highlighted that narrow reflections were indeed expected (Supporting Information Figure 1), and analysis of the reflections reported in Figure 3 using the Scherrer equation predicted an approximate particle size of 26.2 nm. Analysis of the discarded waste solid that precipitated during the reaction showed diffraction patterns consistent with a mixture of the double layered complex hydride Li_3_(NH_2_)_2_I (notably a fast ion conductor),^21^ Zn(NH_3_)_4_I_2_, and unreacted LiNH_2_ (Supporting Information Figure 2). We also note that the pattern does not fit a pattern consistent with ZnO, LiOH, or Li_2_O (Supporting Information Figure 3).

**Figure 4:**
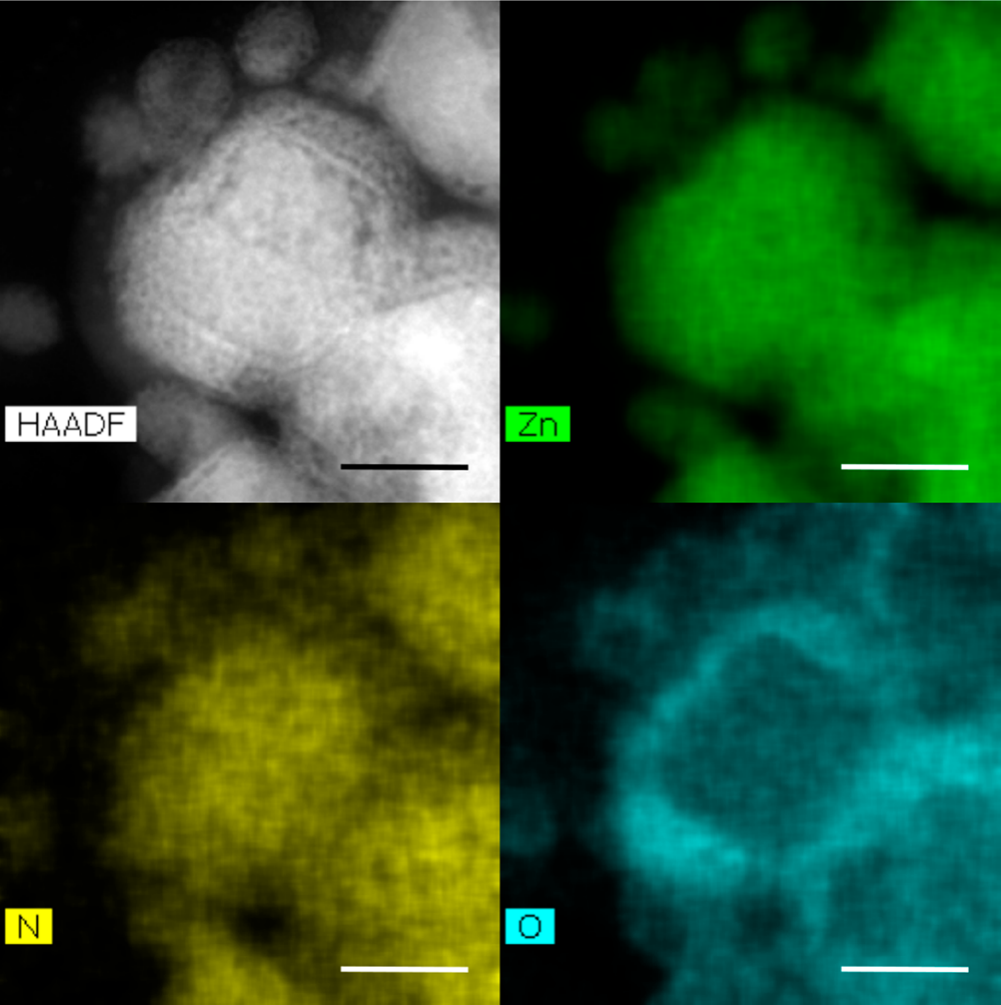
STEM-EDX maps of LiZnN nanoparticles synthesized at 270 °C for 70 min. ADF STEM image (top left) with the corresponding EDX elemental maps of zinc, nitrogen, and oxygen for the region highlighted by in the HAADF image. Scale bar 50 nm.

Elemental mapping of the smaller particles using scanning transmission electron microscopy (STEM) energy dispersive X-ray spectroscopy (EDX) clarified the elemental composition of the core and shell regions of the materials as identified by the Zn, N, and O maps. In Figure 4, a faceted LiZnN particle prepared at 270 °C, imaged using (HAADF)-STEM, is shown, with a clear interface between the core and native inorganic shell. While zinc and nitrogen extend across both core and shell, oxygen is clearly restricted to the shell region. Unfortunately, neither STEM-EDX nor electron energy loss spectroscopy (EELS) was able to confirm the presence of lithium due to the nanoparticles’ sensitivity to the electron beam coupled with the element’s low fluorescent yield and instrumental limitations. The quantified ratio of Zn:O:N recorded in the shell region using STEM-EDX was *ca*. 1:1:1, suggesting that the shelling material was zinc oxynitride, a high-mobility, low-band-gap material composed of Zn_3_N_2_, ZnO, and ZnO_*x*_N_*y*_.^22^ Overall, while considering the optical properties, XRD, TEM, and EDX, the reaction can be summarized as yielding faceted LiZnN/ZnON core/shell particles, *ca*. 20–30 nm in diameter.

To further identify the structures of particles, bright-field and annular dark-field scanning transmission electron microscopy (BF-STEM and ADF-STEM) provided atomic resolution lattice information on the core of the largest nanoparticles, which was consistent with simulated STEM data of [100] orientated Zn_3_N_2_ (Supporting Information Figure 4). Similar analysis of the predominant smaller particles prepared at 270 °C showed atomic resolution images consistent with the crystal structure of LiZnN as shown in the simulation and could not be related to Zn_3_N_2_, ZnO, ZnS, or other oxides (Supporting Information Figure 5). The origin of the oxygen in the shell at this stage is unclear. While oxygen has been detected at the interface of InP and ZnS in InP/ZnS core–shell quantum dots,^23^ this has been attributed to oxygen inherent in the capping agent. In our case, we suggest that although care was taken to maintain an inert atmosphere throughout synthesis and subsequent manipulations, oxygen was included through brief atmospheric exposure either during the purification cleaning process or in the transfer to the electron microscope, highlighting the highly air sensitive nature of this system.

## Conclusions

In conclusion, we have demonstrated lithium insertion into the lattice of Zn_3_N_2_ quantum dots to produce a half-Heusler LiZnN nanoparticle system with a ZnON shell and other Li/Zn-based materials (including larger particles of Zn_3_N_2_) as side-products. This report highlights the rich nitride solid-state chemistry still to be exploited and the diverse structures that exist in nonstandard semiconducting materials that may eventually offer a replacement for traditional binary heavy-metal-based quantum dots.

## Supporting Information Available

Reagents, experimental and analytical techniques, XRD of byproduct, potential products, and simulated diffraction pattern of 20 nm particles. ADF-STEM images and simulated diffraction patterns of nanoparticles (PDF)

## References

[ref1] Tamang S.; Lincheneau C.; Hermans Y.; Jeong S.; Reiss P. Chemistry of InP nanocrystal synthesis. Chem. Mater. 2016, 28 (28), 2491–2506. 10.1021/acs.chemmater.5b05044.

[ref2] McHugh K. J.; Jing L.; Behrens A. M.; Jayawardena S.; Tang W.; Gao M.; Langer R.; Jaklenec A. Biocompatible semiconductor quantum dots as cancer imaging agents. Adv. Mater. 2018, 30, 1706356 10.1002/adma.201706356. 29468747

[ref3] aBrus L. Electronic wave functions in semiconductor clusters: experiment and theory. J. Phys. Chem. 1986, 90, 2555–2560. 10.1021/j100403a003.

[ref4] Taylor P. N.; Schreuder M. A.; Smeeton T. M.; Grundy A. J. D.; Dimmock J. A. R.; Hooper S. E.; Heffernan J.; Kauer M. Synthesis of widely tunable and highly luminescent zinc nitride nanocrystals. J. Mater. Chem. C 2014, 2 (2), 4379–4382. 10.1039/C4TC00403E.

[ref5] Jiang N.; Roehl J. L.; Khare S. V.; Georgiev D. G.; Jayatissa A. H. An *ab initio* computational study of pure Zn_3_N_2_ and its native point defects and dopants Cu, Ag and Au. Thin Solid Films 2014, 564 (564), 331–338. 10.1016/j.tsf.2014.05.032.

[ref6] Zack L. N.; Ziurys L. M. The pure rotational spectrum of ZnS (X^1^Σ^+^). J. Mol. Spectrosc. 2009, 257 (257), 213–216. 10.1016/j.jms.2009.08.009.

[ref7] Carlsson A. E.; Zunger A.; Wood D. M. Electronic structure of LiZnN: interstitial insertion rule. Phys. Rev. B 1985, 32 (32), 1386–1389. 10.1103/PhysRevB.32.1386. 9937172

[ref8] Pereira N.; Klein L. C.; Amatucci G. G. The electrochemistry of Zn_3_N_2_ and LiZnN: A lithium reaction mechanism for metal nitride electrodes. J. Electrochem. Soc. 2002, 149, A262–A271. 10.1149/1.1446079.

[ref9] Kuriyama K.; Kato T.; Tanaka T. Optical band gap of the filled tetrahedral semiconductor LiZnN. Phys. Rev. B 1994, 49 (49), 4511–4513. 10.1103/PhysRevB.49.4511. 10011371

[ref10] Roy A.; Bennett J. W.; Rabe K. M.; Vanderbilt D. Half-Heusler semiconductors as piezoelectrics. Phys. Rev. Lett. 2012, 109, 037602 10.1103/PhysRevLett.109.037602. 22861897

[ref12] Wei S.-H.; Zunger A. Calculated natural band offsets of all II-VI and III-V semiconductors: chemical trends and the role of cation *d* orbitals. Appl. Phys. Lett. 1998, 72 (72), 2011–2013. 10.1063/1.121249.

[ref13] Yoo S.-H.; Walsh A.; Scanlon D. O.; Soon A. Electronic structure and band alignment of zinc nitride, Zn_3_N_2_. RSC Adv. 2014, 4 (4), 3306–3311. 10.1039/C3RA46558F.

[ref14] Walsh A.; Wei S.-H. Theoretical study of stability and electronic structure of Li(Mg,Zn)N alloys: a candidiate for solid state lighting. Phys. Rev. B 2007, 76 (76), 195208 10.1103/PhysRevB.76.195208.

[ref15] White M. A.; Thompson M. J.; Miller G. J.; Vela J. Got LiZnP? Solution phase synthesis of filled tetrahedral semiconductors in the nanoregime. Chem. Commun. 2016, 52 (52), 3497–3499. 10.1039/C5CC09635A. 26839924

[ref16] Men L.; White M. A.; Andaraarachchi H.; Rosales B. A.; Vela J. Synthetic development of low dimensional materials. Chem. Mater. 2017, 29, 168–175. 10.1021/acs.chemmater.6b02906.

[ref17] White M. A.; Miller G. J.; Vela J. Polytypism and unique site preference in LiZnSb: a superior thermoelectric reveals its true colors. J. Am. Chem. Soc. 2016, 138, 14574–14577. 10.1021/jacs.6b10054. 27766839

[ref18] Fairclough S. M.; Taylor P. N.; Smith C. T.; Clark P. C. J.; Skalsky S.; Ahumada-Lazo R.; Lewis E. A.; Tate D. J.; Spencer B. F.; Burkitt-Gray M.; Pis I.; Bondino F.; Bergstrom-Mann P.; Carter-Searjeant S.; Turner M. L.; Binks D.; Haigh S. J.; Flavell W. R.; Curry R. J.; Green M. A. Photo- and electroluminescence from Zn-doped InN semiconductor nanocrystals. Adv. Opt. Mater. 2020, 8, 2000604 10.1002/adom.202000604.

[ref19] Moriga T.; Takahara K.; Saki R.; Sakamoto T.; Murai K.; Nakabayashi I. Synthesis of divalent metal nitrides Zn_3_N_2_ and Mg_3_N_2_ and enhancement of their bandgap by insertion of lithium. Processing and Fabrication of Advanced Materials XIII; Gupta M., Srivatsan T. S., Lim C. Y. H., Varin R. A., Eds.; Stallion Press, 2005; Vol. 1, pp 496–507.

[ref20] Ahumada-Lazo R.; Fairclough S. M.; Hardman S. J. O.; Taylor P. N.; Green M.; Haigh S. J.; Saran R.; Curry R. J.; Binks D. J. Confinement effects and charge dynamics in Zn_3_N_2_ colloidal quantum dots: Implications for QD-LED displays. ACS Appl. Nano Mater. 2019, 2 (2), 7214–7219. 10.1021/acsanm.9b01714. 32118200 PMC7036766

[ref21] Matsuo M.; Sato T.; Miura Y.; Oguchi H.; Zhou Y.; Maekawa H.; Takamura H.; Orimo S.-I. Synthesis and lithium fast-ion conductivity of a new complex hydride Li_3_(NH_2_)_2_I with double-layered structure. Chem. Mater. 2010, 22 (22), 2702–2704. 10.1021/cm1006857.

[ref22] Lee E.; Benayad A.; Shin T.; Lee H.; Ko D.-S.; Kim T. S.; Son K. S.; Ryu M.; Jeon S.; Park G.-S. Nanocrystallie ZnON; high mobility and low band gap semiconductor materials for high performance switch transistor and image sensor application. Sci. Reports 2014, 4, 4948 10.1038/srep04948. PMC4018964 24824778

[ref23] Tessier M. D.; Baquero E. A.; Dupont D.; Grigel V.; Bladt E.; Bals S.; Coppel Y.; Hens Z.; Nayral C.; Delpech F. Interfacial oxidation and photoluminescence of InP-based core/shell quantum dots. Chem. Mater. 2018, 30, 6877–6883. 10.1021/acs.chemmater.8b03117.

