# Supplementary material for: Nanoscale LiZnN - Luminescent Half-Heusler Quantum Dots
Source: ACS Appl Opt Mater. 2023 Jun 6;1(6):1169–73. doi: 10.1021/acsaom.3c00065 (PMC10294247; doi:10.1021/acsaom.3c00065)
Supplement: Supplementary file 1 — ot3c00065_si_001.pdf [file ot3c00065_si_001.pdf]

Supporting Information

Nanoscale LiZnN - Luminescent Half-Heusler Quantum Dots

S. Carter-Searjeant,[a] S. M. Fairclough,[a] S. J. Haigh,[b] Y. Zou,[b] R. J. Curry,[c] P. N. Taylor,[d] C. Huang,[e,g] R. Fleck,[f] P. Machado,[f] A. I. Kirkland,[e,g] M. A. Green [a*]

a) Department of Physics, King's College London, Strand, London WC2R 2LS, UK.

*Corresponding author (mark.a.green@kcl.ac.uk)

b) Department of Materials, University of Manchester, Oxford Road, Manchester M19 9PL, UK.

c) Department of Electrical and Electronic Engineering, Photon Science Institute, University of Manchester, Oxford Road, Manchester M13 9PL, UK.

d) Sharp Life Science (EU) Ltd. The Hayakawa Building, Edmund Halley Road, Oxford Science Park, Oxford OX4 4GB, UK.

e) Electron Physical Sciences Imaging Centre, Diamond Light Source, Harwell Science Innovation Campus. Fermi Ave, Didcot, OX110DE, UK.

f) Centre for Ultrastructural Imaging, New Hunts House, Guys Campus, King's College London, London, SE1 1UL, UK.

g) Department of Materials, University of Oxford, Parks Road, Oxford OX1 3PH, UK.

Materials and chemicals

All chemical reagents were purchased from Sigma Aldrich. 1-octadecene (ODE, 95%), zinc iodide (ZnI_2, 95%), lithium amide ($LiNH_2$, 95%), 1-hexadecanethiol (HDT, 95%), cyclohexane (99.5%, anhydrous), isopropanol (solvent, 99.5%, anhydrous) and ethanol (99.5%, anhydrous).

Reagent Preparation

1-octadecene (ODE) was placed under vacuum at 100-110 °C for 30 minutes and then cooled and kept under an N_2 atmosphere. All reagents/solvents were degassed with nitrogen using a Schlenk line for 30 mins before being stored in the glovebox.

Synthesis

Zinc iodide (143.3 mg, 0.45 mmol), lithium amide (219.6 mg, 9.6 mmol), hexadecanethiol (200 µl), and 1-octadecene (15 ml) were placed in a 3-neck round vessel in a glovebox under nitrogen and sealed, forming a white cloudy suspension. The system was transferred to a Schlenk line under positive N_2 pressure, and the atmosphere was purged for a further 30 minutes. The system was then heated to 250 °C under vigorous stirring under a closed system. The decomposition phase occurred at 190 °C, where the solution effervesced for a few minutes. Yellow, green, brown, black solutions were produced with increasing time. The solution was then heated to 270 °C for a total of 70 minutes to make a red solution with red precipitate falling to the bottom of the flask. After the reaction had finished, the flask was removed from the heat and allowed to cool to room temperature.

Purification

The solution was cleaned in a glovebox, by adding 1:1 ratio [nanocrystal solution: cyclohexane] and a 1:1 ratio of [nanocrystal solution: ethanol], which was centrifuged at 5000 rpm for 5 minutes. The supernatant was discarded with pellet remaining. This cleaning processes was repeated twice.

Finally, the pellet could then be dispersed in cyclohexane (1 mL), for TEM, STEM, PLQY, EDXS, emission and UV absorption analysis. For XRD analysis the sample was remained as a pellet form and was dried under vacuum.

Characterisation

Emission Spectroscopy

For the photoluminescence studies, cleaned/purified samples were excited using a 395 nm ThorLabs diode laser with emission detected using a Horiba FluoroMax4 modular spectrophotometer and an Ocean Optics QE Pro spectrometer with 415nm band-pass filter. All cleaned samples were analysed in air-tight (with screw caps) 1cm path-length Quartz cuvettes. Samples were purified and prepared in a glove box, to prevent oxidation. The screw caps were para-filmed and placed in a nitrogen filled box during transportation to the measurement apparatus. The parafilm was removed prior to analysis. Data was analysed using Origin software.

UV-Absorption Spectroscopy

Absorption spectra were measured by using a Hitachi U-4100 UV-vis-NIR spectrophotometer, with cyclohexane as a reference sample. The cleaned samples were analysed in air-tight quartz cuvettes, prepared in a glove box. The screw caps were para-filmed and placed in a nitrogen filled box during transportation to the machinery. The para-film was removed prior to analysis. Data was analysed using Origin software.

Photoluminescence Quantum Yield measurements (PLQY)

For quantum yield measurements, samples were excited using a 405 nm Thorlabs diode laser within a Newport 3.3" integrating sphere and the resultant flux was measured with a calibrated Newport 818-SL silicon photodetector or Ocean optics compact spectrometer. Cyclohexane was used as a reference sample, with all samples in air-tight Quartz cuvettes, as previously mentioned (1). Data was analysed using Origin software.

Transmission Electron Microscopy (TEM)

Transmission electron microscopy was carried out using cleaned samples (in cyclohexane). These were drop cast on Agar Scientific ultra-thin holey carbon support on a Cu 400 mesh grid, all completed in a glovebox. The samples were allowed to evaporate/dry the solvent off the grid, in the glove box before the TEM grids were wrapped in filter paper and placed into nitrogen filled vials/containers ready for transportation. Initial TEM data used JEOL-JEM 1400 at ~90 kV with a

FEI Tecnai T20 camera. Images were analysed in ImageJ software, where average diameter sizes were calculated. Using the same methods as above, later TEM images were taken on the JEOL-JEM-F200 at 120kV with a Gatan Orius CCD camera.

High Resolution Scanning Transmission Electron Microscopy (STEM)

High Angle Annular Dark Field STEM images, EDX and Electron Energy Loss Spectroscopy (EELS) measurements were taken on FEI Titan G2 ChemiSTEM at 200kV. High atomic resolution STEM images were taken on a JEOL ARM 300CF. Grids were loaded in a glovebox and placed into a vacuum transfer holder to prevent oxidation during transfer of the grids from the glovebox to the microscope.

Energy dispersive X-ray Spectroscopy (EDS)

EDS maps were collected with a Titan (SuperX) 4 SDD with total collection angle of 0.7 sr. The total image collection time was between 2 and 15 minutes, (FEI Titan G2 ChemiSTEM). EDS data was also obtained using an Oxford instruments 80 mm thin-window EDS detector with a 0.28 sr collection angle (XEDX system) (JEOL ARM 300CF).

X-Ray Diffraction (XRD)

Powder X-Ray diffraction was carried out using a Mo source-kα Stoe STADI-P diffractometer using thin-foil mode, molybdenum X-ray source at 0.7093 Å, 50 kV, and 30 mA with a 2 theta scan 2.000° to 40.115° step 0.495° at 30.0 s/step. Before analysis, under a nitrogen atmosphere, samples were centrifuged down to a small solid (black/red) pellet (5000rpm for 5mins (x 2)). Samples were left under vacuum overnight to dry. Later the sample was placed in the glovebox and left to dry out under N_2 for 1 hour. In the glovebox, the samples were crushed using a spatula and place on Kapton tape. 1mm thick Kapton tape (formally known as polyoxydiphenylene-pyromellitimide, a polyimide, supplied by BERTECH) was used to analyse and compact the solid samples. XRD reference patterns were collected from the Crystallography Open Database (COD) and the International Centre for Diffraction Data (ICDD). QualX2.0 and EVA database (Bruker, U.S.) were used for sample identification.

Analysis software

Data analysis was carried out using Origin software for emission, absorption and XRD data. STEM/TEM images were calculated using Image J and Gatan Digital micrograph software. EDXS spectra were analysed with Hyperspy and element maps were determined through Bruker software.

References

1. De Mello, J C, H F Wittmann and R H Friend. "An improved experimental determination of external photoluminescence quantum efficiency." *Adv. Mater.*, **1997**, (9), 230-232.

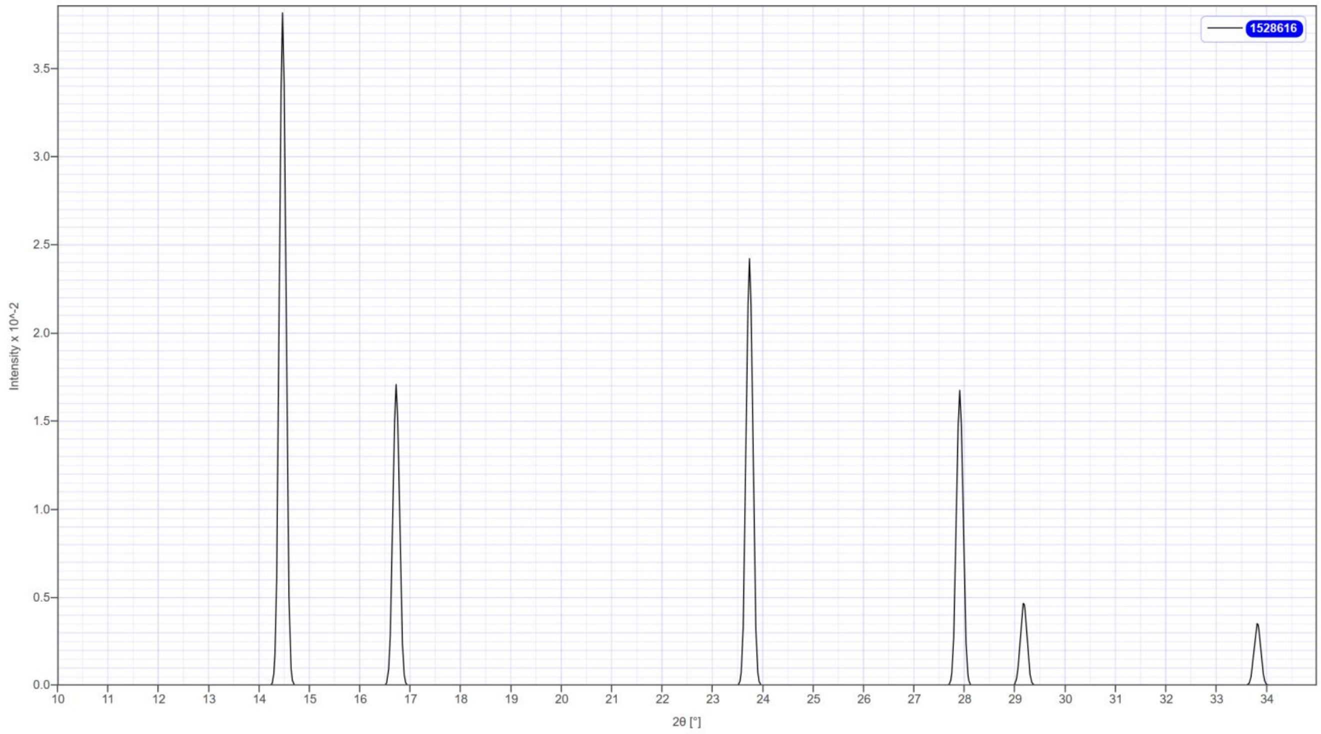

Figure S1. The simulated reflections from 22 nm LiZnN particles using CrystalMaker software.

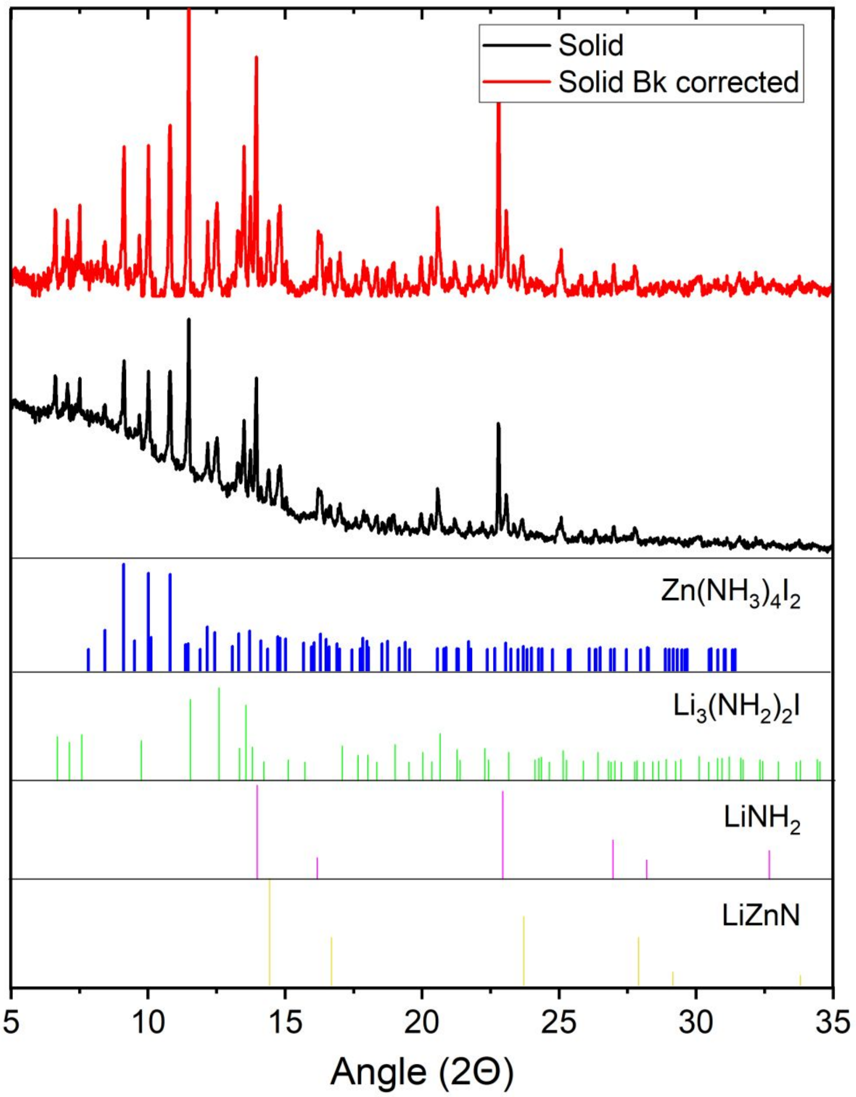

Figure S2. XRD spectra of crude solid (black line), with reference data over-laid. $Zn(NH_3)_4I_2$ (blue line), Lithium diamide lithium iodide (green line) and $LiNH_2$ (pink line) and LiZnN (orange line).

Reference data can be found using [PDF card 001-0792], [PDF card 012-0688], [PDF card 035-0762], [PDF card 038-0356], [PDF card 043-1929], [PDF card 046-0943], [PDF card 050- 0412], [PDF card 052-0976].

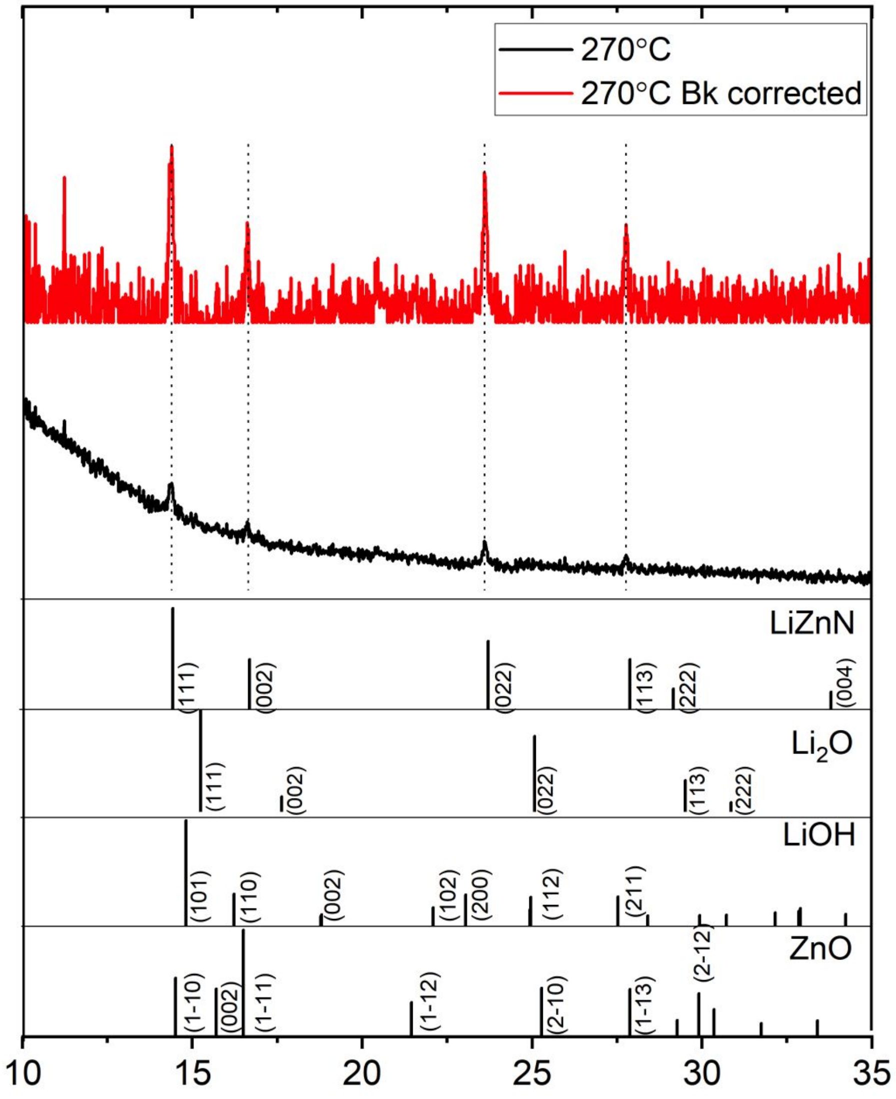

Figure S3. XRD spectra of crude solid (black line), with reference data for potential side products Li_2O, LiOH, and ZnO.

Reference data can be found using [PDF Card - 00-032-0564], [PDF card - 00-036-1451] and [PDF card - 00-012-0254].

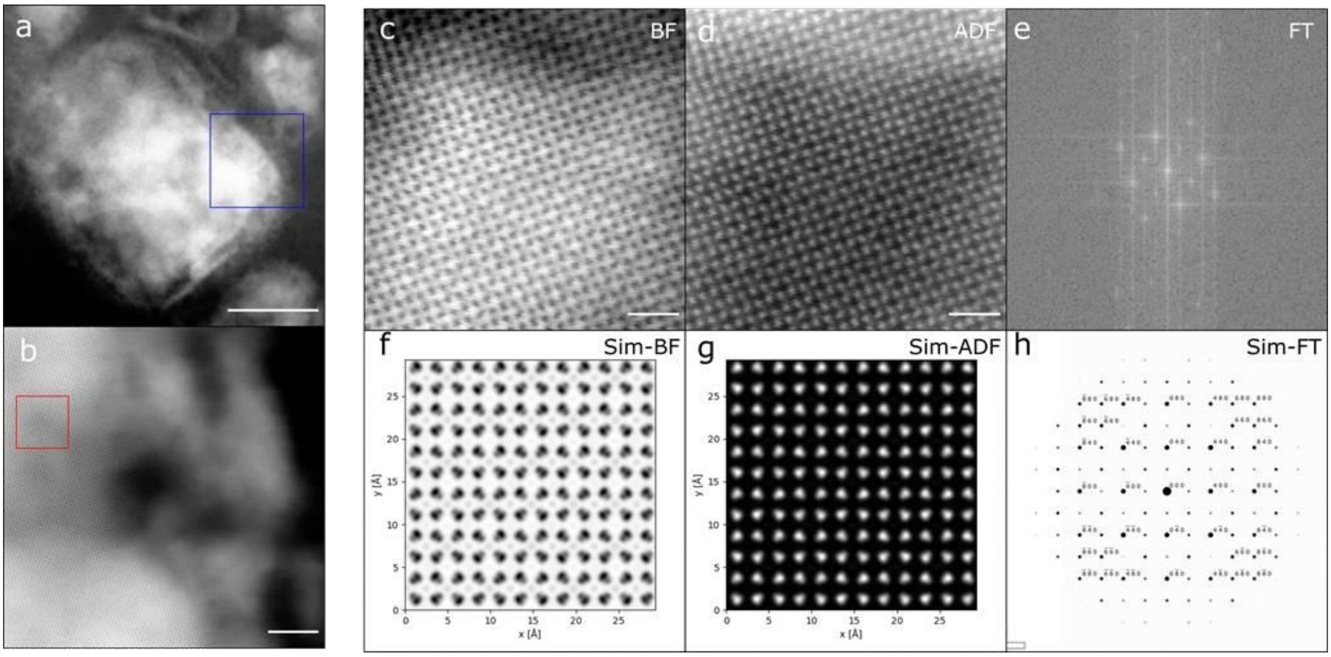

Figure S4 (a-b) ADF-STEM images of a large square nanoparticle prepared at 270°C highlighting a mottled surface, (c-d) shows a high magnification high resolution. (e) The power spectrum of figure d. **(c)** bright field (BF) and **(d)** annular dark-field (ADF) micrographs at the centre of the particle. (f,g,h) Simulated (100) orientated Zn_3N_2 (f), BF,(g) DF and (h) simulated diffraction pattern, supporting Zn_3N_2 core particle. Scale bars a) 20nm, b) 5nm, c) 1nm, d) 1nm.

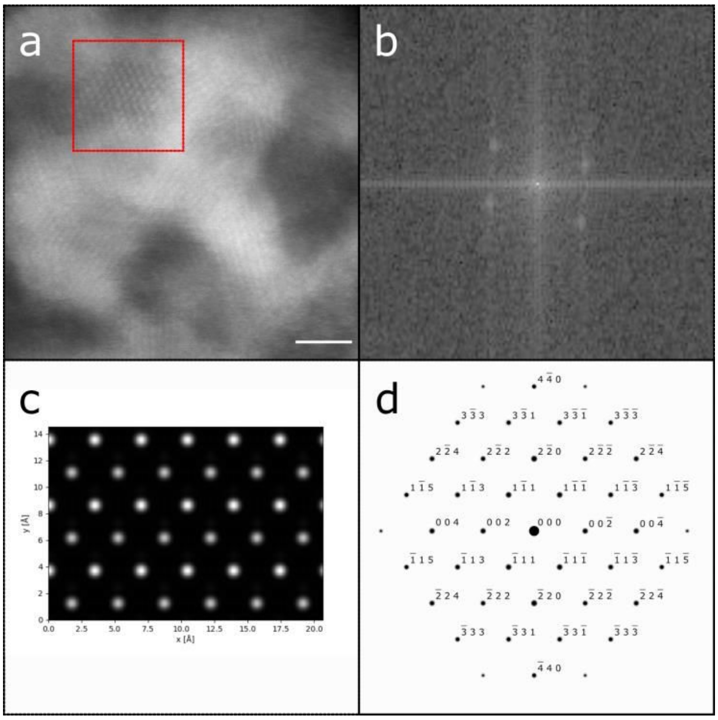

Figure S5 a) ADF-STEM image of the core of smaller nanoparticle prepared at 270°C. b) the corresponding Fourier transform of the red region consistent with the (110) LiZnN as shown by simulation. c) ADF and d) simulated diffraction patternd. (a) Scale bar 2nm.
